# Photosynthetic performance and carbon metabolism in the ear organs of oats under drought stress

**DOI:** 10.3389/fpls.2024.1463284

**Published:** 2025-01-21

**Authors:** Jiaqi Fang, Yuan Zhan, Baowen Zhao, Yan Zhao, Youjun Chen, Qingping Zhou, Hui Wang

**Affiliations:** ^1^ Sichuan Zoige Alpine Wetland Ecosystem National Observation and Research Station, Southwest Minzu University, Chengdu, China; ^2^ Bijie City Control Rocky Desertification Management Center, Bijie Forestry Bureau, Bijie, China

**Keywords:** photosynthetic contribution, non-leaf organs, glume, carbohydrate content, ear organs

## Abstract

Sufficiently exploiting the potential of crop photosynthesis is one of the critical ways for improving cultivation production to face global climate change. In this study, oat plants were potted with three watering treatments. The glumes, lemmas, and flag leaves were sampled on days 0, 7, and 14 after the first floret blossomed under the control (denoted as CK-0, CK-7, and CK-14), drought stress (denoted as DS-7, and DS-14) and rewatering treatments (RW-14). Paraffin cross-section structures were observed, and the absolute water content, photosynthetic enzyme activities, carbohydrate content, dry matter weight, and total C and total N accumulation were determined in the glumes, lemmas and flag leaves. The results showed that stomatal tissues were present in both the inner and outer epidermis in the glumes and lemmas, and chloroplasts existed in the cells of both ear organs. Compared to CK-14, the absolute water content was significantly decreased in the flag leaves, stems, and seeds under DS-14, while drought stress did not significantly affect the water status of the glumes, lemmas, and peduncles. Drought stress significantly decreased the PEPC activities in the glumes, lemmas, and flag leaves, and the glumes had significantly higher PEPC activity than the flag leaves in the late stages of grain filling. Compared to CK-7, fructose and sucrose content was significantly decreased in the flag leaves under DS-7, while drought stress significantly increased the fructose, sucrose, and starch content in the glumes and lemmas. In addition, soluble sugar content was significantly increased in all glumes, lemmas, and flag leaves under drought stress. Rewatering significantly increased the carbohydrate content in the flag leaves, while it had no significant effect on the glumes and lemmas. As growth continued, the C and N contents and the dry matter mass in the seeds gradually increased, which was transferred from the glumes, lemmas, leaves, and stems. The results suggest that oats can tolerate a certain degree of drought without affecting the ears’ physiological function and yield, and ear organs can maintain water status and photosynthetic performance, which plays a major role in the maintenance of seed yield under drought stress conditions.

## Introduction

Oat *(Avena sativa* L.), an annual cereal, is an important grain-forage crop. Derived from oat grains, β-glucan has been shown to offer a variety of health benefits such as reducing weight (Mathews et al., 2023), lowering glucose (Regand et al., 2009) and lipid levels (Wolever et al., 2010), improving cardiovascular health (Earnshaw et al., 2017), and boosting immunity. As a forage crop, immature oat plants are characterized by their high sugar content, high relative feeding value, high biomass yield, good palatability, and high digestibility (Kuter et al., 2023). The oat is mainly planted in Europe, Northern America, and Asia ranking sixth in terms of the global cultivation area of cereal crops according to the Food and Agriculture Organization (FAO, 2024). China, being one of the world’s largest oat producers and consumers, extensively cultivates oats in its northern, northwestern, and southwestern areas. However, the climate in Northern and Northwestern China is consistently dry with minimal rainfall making water one of the most crucial environmental factors limiting the growth and production of oats.

Droughts disrupt plants’ internal physiological activities, such as photosynthesis, reducing plant metabolism and inducing premature senescence, leading to reduced production (Morales et al., 2020). Typically, we consider leaves as the primary organ for photosynthetic carbon fixation in plants. Previous studies have shown that drought stress causes accelerated degradation of photosynthetic components (chlorophyll, Rubisco, photosystem II, etc.) in the flag leaves of cereal crops (Tian et al., 2022; Juzoń et al., 2020; Ghotbi-Ravandi et al., 2014; Martinez et al., 2003). In addition, light trapping, ATP synthesis, and key genes involved in the dark reaction are repressed under drought stress (Vicente et al., 2018) decreasing the rate of photosynthesis. However, further research has shown that the sensitivity of various plant organs to drought stress varies significantly. When exposed to drought stress, plants’ reproductive organs tend to senesce later (Brazel and Ó Maoiléidigh, 2019; Kong et al., 2015) and maintain a more stable physiological state than their leaves (Sanchez-Bragado et al., 2020). For cereal crops like oats, the ear grows at the top of the plant providing them an advantage over the leaves in terms of capturing light and CO_2_ (Sanchez-Bragado et al., 2020). In the ears, the inner epidermal stomata of the glumes rapidly remobilize CO_2_ produced via grain respiration (Araus et al., 1993), which also facilitates the transfer of photosynthate from the spikes to the grains more quickly and with lower losses (Sanchez-Bragado et al., 2014). Additionally, the thick dorsal epidermis and cuticle of ear organs, such as glumes, lemmas, and awns, contribute to reducing water evaporation and protecting photosynthetic gas exchange during drought stress (Sanchez-Bragado et al., 2020; Martinez et al., 2003; Araus et al., 1993).

Carbohydrates, the main photosynthetic products, provide energy and carbon skeletons for plants with various types of metabolism, but they are highly influenced by water status. The performance of carbohydrate transport and metabolism reflects the strength of plant resistance (Zahoor et al., 2017). Carbohydrates can be categorized into two types based on the form in which they exist. Among them, non-structural carbohydrates are the main reserve substances for plant life activities and key regulators of plant resistance to abiotic stresses (Valluru et al., 2011). Sucrose is the primary form of transportation for non-structural carbohydrates in plants (Ullah et al., 2023). Starch is the primary form of storage for photosynthetic products in plants and serves as the primary carbon source for grain filling in the later stages of fertility. The decrease in causal seed yield that occurs during drought stress is mainly due to the limitation of starch synthesis (Ahmed et al., 2020). Under drought stress, plants enhance their resistance to drought by synthesizing large amounts of carbohydrates and using these carbohydrates as osmotic agents to regulate the osmotic potential of their cells (Rao and Chaitanya, 2016). However, there are still numerous shortcomings in the study of oat carbon metabolism under drought stress. In addition to examining physiological response mechanisms, we can also direct our focus toward the molecular response mechanisms of ear organs. By integrating these two mechanisms, we can conduct a comprehensive analysis to delve deeper into the carbon metabolism of ear organs under drought stress.

This study primarily explores the physiological responses of oats (*Avena sativa* L.) to drought stress, with a particular emphasis on the mechanisms involved in photosynthesis, carbohydrate metabolism, and plant drought tolerance. By investigating how drought impacts the key components of photosynthesis in oat flag leaves and spikes (such as photosynthetic enzymes and CO_2_ response curves), we revealed the detrimental effect of drought on photosynthetic rate, while highlighting the spike organs’ advantage in tolerating drought stress. We compared the physiological states of flag leaves and spike organs under drought conditions and explored the distinct responses of these organs to drought stress. Furthermore, we analyzed how drought influences the accumulation of non-structural carbohydrates, such as sucrose and starch, in different oat organs, as well as the implications of these changes for plant stress tolerance. Through this study, we aim to gain a deeper understanding of the physiological adaptation mechanisms of oats under drought conditions highlighting the tolerance capabilities of spike organs in response to drought environments. This provides a scientific basis and insights for improving oat drought tolerance and enhancing agricultural management practices.

## Materials and methods

### Experimental design

*Avena sativa* L. cv. Taike seeds were sown in plastic pots (height = 22 cm, diameter = 23 cm) in the experimental region of Southwest Minzu University (103°58′10.758″E, 30°33′42.455″N), in Chengdu City, Southwestern China, in November 2022. The average temperature from November to April in Chengdu during the experimental period is 8°C–20°C. Each pot was filled with a soil weight of 5 kg, containing a 3:1 mixture of nutrient soil/vermiculite. The nutrient soil was described with pH 5.5, EC 20 mS/m, NO_3_–N 64.23 g/m^3^, NH_4_–N 28.90 g/m^3^, and P_2_O_5_ 76.83 g/m^3^. Ten seeds were sown in each pot. Seedlings were thinned at the three-leaf stage, and six healthy plants with uniform growth were kept in each pot. At the tillering stage, 500 ml of modified Hoagland’s complete nutrient solution was applied to each pot, and at the jointing stage, 1.6 g of urea (containing 46% N) was applied to each pot. Watering during the nutrient growth stage was maintained at 70%–80% of the maximum soil water holding capacity. During the plant heading stage, a transparent plastic sheet was used to construct a rain shelter 2.5–3 m above the planting area to protect the plants from rain. The main reproductive branch per plant was tagged. Water stress treatments were carried out when the first floret blossomed in the pots and samples were collected as CK-0. After 7 days of treatment, samples were collected as CK-7 and DS-7. Following that, some of the DS-7 samples were resumed with watering (RW treatment). After another 7 days, samples were collected as CK-14, DS-14, and RW-14. Watering was maintained at 35%–45% of the maximum soil water holding capacity for the water stress group (DS), while soil water content was kept at 70%–80% for the control group (CK). The water content of the rewatering group (RW) was restored to 70%–80% after 7 days of drought treatment. Totally, 52 pots were sowed in this study including 23 pots for CK and DS treatment, respectively, and six pots for RW treatment.

### Paraffin section preparation and observation

The paraffin sections were produced and scanned in accordance with the methods reported in the process reference (Soukup and Tylová, 2014). The paraffin sections of CK-0, CK-14, and DS-14 were placed under an upright light microscope (DS-U3, Nikon, Japan) for observation, and image acquisition was completed.

### Absolute water content

The main reproductive branches of oats from each treatment were collected with three repetitions at 6:00 p.m. on days 0, 7, and 14 after the first floret blossomed under the CK (denoted as CK-0, CK-7, and CK-14, respectively), DS (denoted as DS-0, DS-7, and DS-14, respectively), and rewatering treatments (denoted as RW-14). Stems (with leaf sheaths), flag leaves, peduncles, glumes, lemmas, and seeds were promptly separated and weighed to determine their fresh weight (M1). Each part was then placed in a separate envelope bag and subjected to 105°C for 30 min. Then, they were subjected to 65°C for drying until a constant weight (M2) was achieved (Song et al., 2022). The absolute water content (AWC) of each part was then calculated as follows:


AWC (%)=((M1 − M2)/M1) ×100%

### Light and CO_2_ response curve

The gas exchange rates of the flag leaves, glumes, lemmas (including the floret or embryo), and ears of the main reproductive branches were measured when the first floret blossomed using a portable photosynthesizer (Li-6800, LI-Cor, United States) at 9:00–11:00 a.m. with three repetitions (Tian et al., 2022). We chose a gasket with an air chamber measuring 2 cm², and the light intensity of the gas chamber was first adjusted to 1,500 µmol m−² s−¹. After gas exchange stabilized, automatic measurement mode of light curve was applied with light intensities of 2,100, 1,800, 1,500, 1,200, 900, 600, 300, 200, 150, 100, 70, 30, and 0 µmol m−² s−¹. Each light intensity gradient was maintained for 3 min. The CO_2_ concentration gradients were set to 400, 300, 200, 100, 50, 0, 200, 400, 600, 800, 1,000, and 1,200 µmol m−¹. At the end of the measurements, the plant tissues were cut inside the gas chamber and weighed to determine their fresh weight. The photosynthetic rate is calculated by dividing the gas exchange rate by the fresh weight of a sample.

### Photosynthetic enzyme activities

Flag leaves, glumes, and lemmas from the main reproductive branches were collected from each treatment including CK-0, CK-7, CK-14, DS-7, DS-14, and RW-14. The samples were stored at −80°C to measure Rubisco and PEPC activities, and the measurements were replicated three times. The crude enzymes of Rubisco and PEPC were extracted according to the kit instructions (Suzhou Comin Biotechnology Co., China). Subsequently, the crude enzyme extracts were added to 96-well plates along with the necessary working reaction solution (Tian et al., 2022). The absorbance values A1 at 20 s and A2 after 5 min at 340 nm were promptly measured using an enzyme labeler (Varioskan LUX, Thermo Scientific, United States).


$$\begin{array}{c} Rubisco\;activity\text{ (}nmol/min/g\;fresh\;weight\text{) }\\ =\;1,286\;*\text{ (}A1\;-\;A2)/M \end{array}$$

$$\begin{array}{c} PEP\;activity\text{ (}nmol/min/g\;fresh\;weight\text{) }\\ =\;1,286\;*\text{ (}A1\;-\;A2)/M \end{array}$$

In these equations, M is the fresh weight of the sample (g).

### Carbohydrate content

At 7:00 a.m., 1:00 p.m., and 8:00 p.m. 0.1 g of flag leaves, glumes, and lemmas from the main reproductive branches of each treatment, including CK-0, CK-7, CK-14, DS-7, DS-14, and RW-14, was immediately frozen in liquid nitrogen, and this process was replicated three times. The quickly frozen samples were processed using a high-throughput tissue grinder (SCIENTZ-48, Ningbo Xinzhi Biotechnology Co. Ltd., China) for grinding. After being ground, the samples were stored in liquid nitrogen. The content of carbohydrates, such as fructose, sucrose, starch, and soluble sugars, was measured using the kits obtained from Suzhou Comin Biotechnology Co (Jin et al., 2022).

### Dry matter mass

One main reproductive branch of oat was chosen from each of the four pots used, and the ears were shaded with aluminum foil tied with a 1-mm aperture (recorded as CK-se and DS-se) until seed maturity. Additionally, four main reproductive branches of oats were randomly selected and subjected to glume removal treatment (recorded as CK-rg and DS-rg). Once the seeds had matured, the stems (including leaf sheaths), flag leaves, peduncles, glumes, lemmas, and seeds of the plants corresponding to CK-se, DS-se, CK-rg, and DS-rg were separated (Wei et al., 2023). These samples were then dried at 65°C for 20 h and weighed. The photosynthetic contribution was calculated as follows:


$$Photosynthetic\;contribution\;of\;ear\;=\;\left(SY1\;–\;SY2\right)/SY1.$$

$$Photosynthetic\;contribution\;of\;glumes\;=\;\left(SY1\;-\;SY3\right)/SY1.$$

where SY1, SY2, and SY3 are the seed yields of the main reproductive branches with no treatment, with ear shading, and with glume removal, respectively.

### Total C and N content

The samples of each organ were taken after determining the AWC and dry matter mass at seed maturity (for which there were three replications). The samples were ground separately using a high-speed grinder (Tissuelyser-48, Jingxin, China), and the total C and N proportions of each part were analyzed with an elemental analyzer (UNICUBE, Elementar, Germany) (Sun et al., 2013).

### Statistical analysis

Data entry and calculations were organized using Microsoft Excel 2020. Multiple comparisons of the experimental data were conducted using one-way analysis of variance (ANOVA) with the Duncan test using IBM SPSS Statistics 20, and the results were plotted using Origin 2021.

## Results

### Paraffin cross-section structure

**Figure 1:**
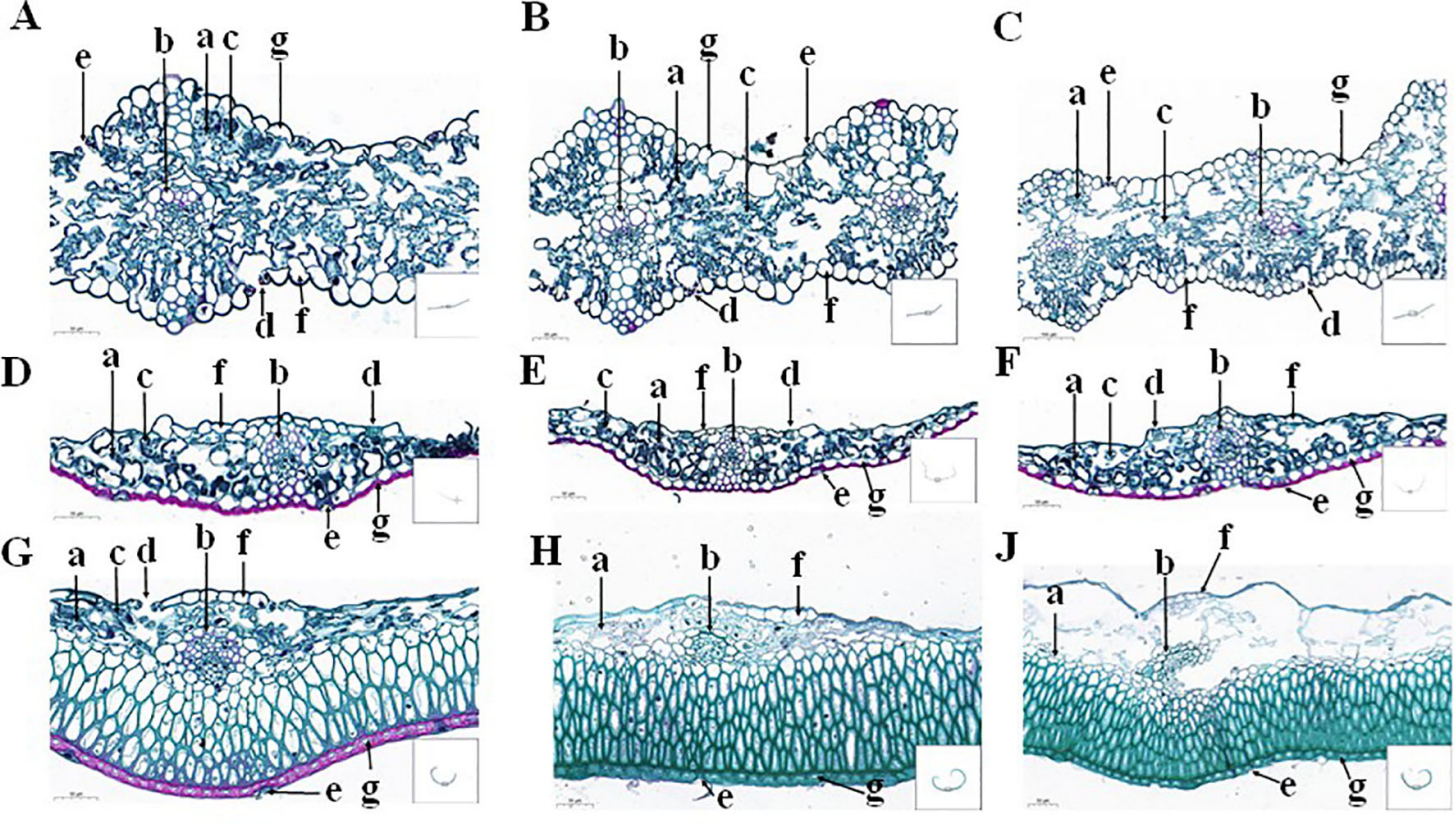
The paraffin cross-sectioned structure of the flag leaves **(A–C)**, glumes **(D–F)**, and lemmas **(G, H, J)** under the CK-0 **(A, D, G)**, CK-14 **(B, E, H)**, and DS-14 **(C, F, J)** treatments. a, histiocytes; b, bundle sheath cells; c, chloroplasts; d, inner epidermal stomata; e, outer epidermal stomata; f, inner epidermal cells; g, outer epidermal cells.

The upper and lower epidermis of the CK-0 flag leaves had a complete and mature stomatal organization. The spongy tissue was irregularly dispersed within the mesophyll cells and contained a large number of chloroplasts. Their vascular bundle sheath cells contained lignified ducts, and keratinized stone cells were observed in the leaf veins (**Figure 1A**). The spongy tissues in the CK-14 flag leaves were atrophied, and the chloroplasts had become smaller (**Figure 1B**). The corkification of stone cells on both sides of the bundle sheath cells increased. However, the spongy tissues in the flag leaves under the DS-14 treatment were more atrophied, and their chloroplasts were more crumpled than those under CK-14 (**Figure 1C**).

The glumes under the CK-0 treatment contained stomatal tissue in both the inner and outer epidermis. Most stomata in the outer epidermis were incomplete (developmentally restricted or degraded), while the stomatal tissue in the inner epidermis was more complete and more standardized. The vascular sheath cells exhibited horseshoe-shaped lignification resembling the Casparian strip. The outer epidermis was externally thickened and had undergone corkification. The lateral tissue cells of the outer epidermis were regularly shaped and neatly arranged, with a large number of chloroplasts (**Figure 1D**). There was no significant change in the glume structure under the CK-14 treatment, but there was an increase in chloroplast numbers and a deepening of the horseshoe-shaped thickening of bundle sheath cells compared to CK-0 (**Figure 1E**). The glume structure under the DS-14 treatment did not change significantly from that under CK-14 (**Figure 1F**).

Under the CK-0 treatment, it was observed that the lemma’s inner and outer epidermis had stomata. The inner epidermal stomata were mainly distributed near bundle sheath cells, while the outer epidermal stomata were similar to or more degraded than the glumes. The outer epidermal cell walls were thickened and had undergone corkification, containing chloroplasts, while the inner epidermis cells were more elongated, lighter, and thinner with no chloroplast (**Figure 1G**). Parenchyma cells in the lemmas under the CK-14 treatment were found to be atrophied, and the chloroplasts were degraded (**Figure 1H**). Under drought stress, the parenchyma cells of the lemma were more severely degraded, and chloroplasts were largely absent (**Figure 1J**).

### Absolute water content

**Figure 2:**
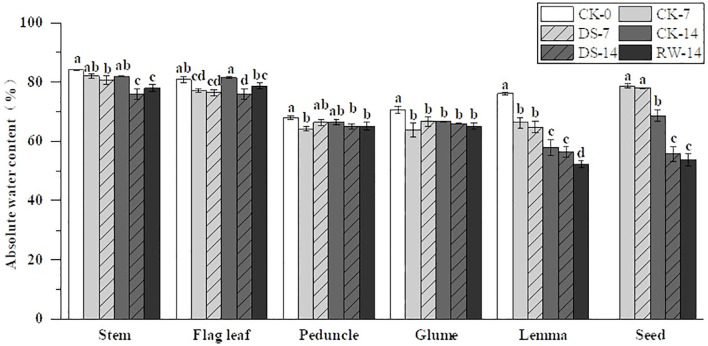
Absolute water content (AWC) in the stems, flag leaves, peduncles, glumes, lemmas, and seeds under different watering treatments at different stages. Different lowercase letters within the same organ indicate a significant difference at the p < 0.05 probability level.

Under drought stress, the AWC of stems, flag leaves, lemmas, and seeds showed varying degrees of reduction (**Figure 2**). Specifically, the AWC of stems, flag leaves, and seeds was significantly (p < 0.05) lower under the DS-14 treatment than under CK-14. However, the impact of drought stress on the AWC of peduncles, glumes, and lemmas was not significant. Compared to CK-14, the AWC of stems, flag leaves, lemmas, and seeds was significantly lower under RW-14. The AWC of the stems and seeds under RW-14 did not differ significantly from that of the stems and seeds under DS-14. However, the AWC of flag leaves under RW-14 was significantly higher than that under DS-14. Rehydration after drought did not have a significant effect on the AWC of both peduncles and glumes.

### Photosynthetic enzyme activities

**Figure 3:**
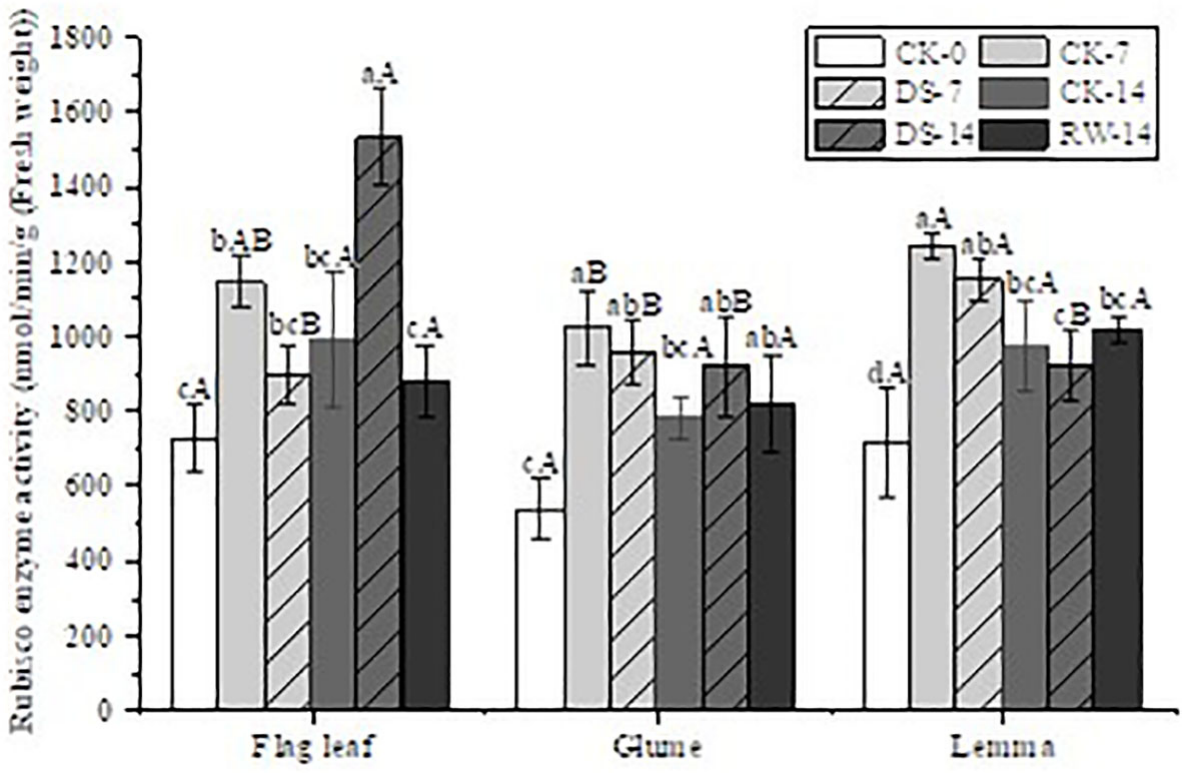
Rubisco enzyme activity in flag leaves, glumes, and lemmas under different watering treatments at different stages. Different lowercase letters within the same organ and different capital letters within the same treatment indicate a significant difference at the p < 0.05 probability level.

Under drought stress, the Rubisco activity in the flag leaves, glumes, and lemmas showed varying decreases under DS-7 compared to that under CK-7 (**Figure 3**). The Rubisco activity of flag leaves under DS-14 was significantly (p < 0.05) higher than that under both CK-14 and RW-14, while no significant difference was found among glumes and lemmas under CK-14, DS-14, and RW-14.

**Figure 4:**
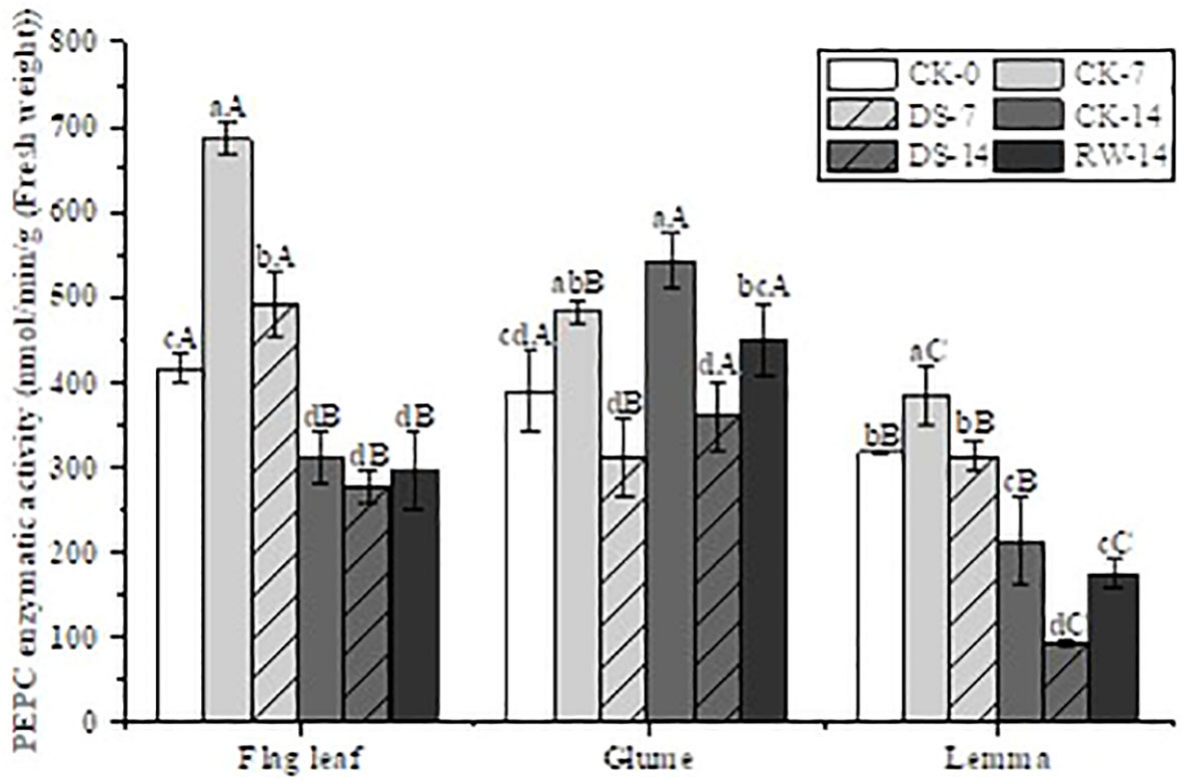
PEPC enzyme activity in flag leaves, glumes, and lemmas under different watering treatments at different stages. Different lowercase letters within the same organ and different capital letters within the same treatment indicate a significant difference at the p < 0.05 probability level.

The PEPC enzyme activity of all three organs was significantly lower (p < 0.05) under DS-7 than under CK-7 (**Figure 4**). Compared to CK-7, the PEPC enzyme activity of both flag leaves and lemmas was significantly lower (p < 0.05) under CK-14, but that of the glumes had not significantly changed. A similar trend was found under the DS-7 and DS-14 treatments as well. Compared to DS-14, the PEPC enzyme activity of both glumes and lemmas under CK-14 and RW-14 was significantly higher (p < 0.05).

### Light and CO_2_ response curves

**Figure 5:**
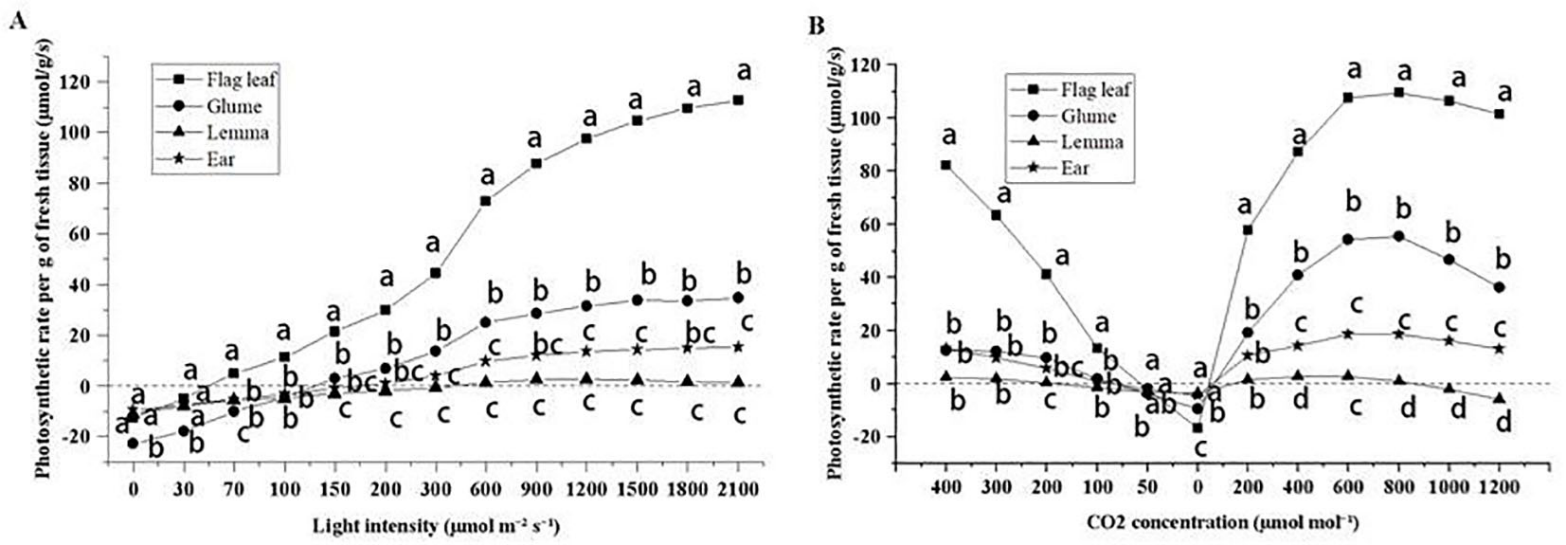
The light response curve **(A)** and CO_2_ response curve **(B)** of flag leaves, glumes, lemmas, and intact spikelets of CK-0. Different lowercase letters within the organ under the same light intensity or CO_2_ concentration indicate a significant difference at the p < 0.05 probability level.

When the light intensity was 0, the gas exchange performance indicated the strength of respiration in photosynthetic organs. As the light intensity increased, the photosynthetic rate also increased in all organs (**Figure 5**). The flag leaves reached the light compensation point first, followed by the glumes and ears, and finally the lemmas. The fastest increase in photosynthetic rate for each organ was observed at light intensities of 300–600 µmol m^−2^ s^−1^. The maximum photosynthetic rate per unit weight of each organ corresponded to the following ranking: flag leaves > glumes > ears > lemmas. Lemmas exhibited photoinhibition after the light intensity exceeded 1,200 µmol m^−2^ s^−1^.

As the CO_2_ concentration decreased, lemmas were the first to reach the CO_2_ compensation point (**Figure 5**). Glumes and ears had similar CO_2_ compensation point, while flag leaves had the lowest compensation points. When the CO_2_ concentration was reduced to 0 µmol m^−1^, the lowest photosynthetic rate was observed in flag leaves followed by glumes. Lemmas and ears exhibited the highest and similar rate. Lemmas were the first to show inhibition upon increasing the amount of CO_2_ supplemented. Flag leaves, glumes, and ears had similar CO_2_ inhibition concentrations.

### Carbohydrate content

**Figure 6:**
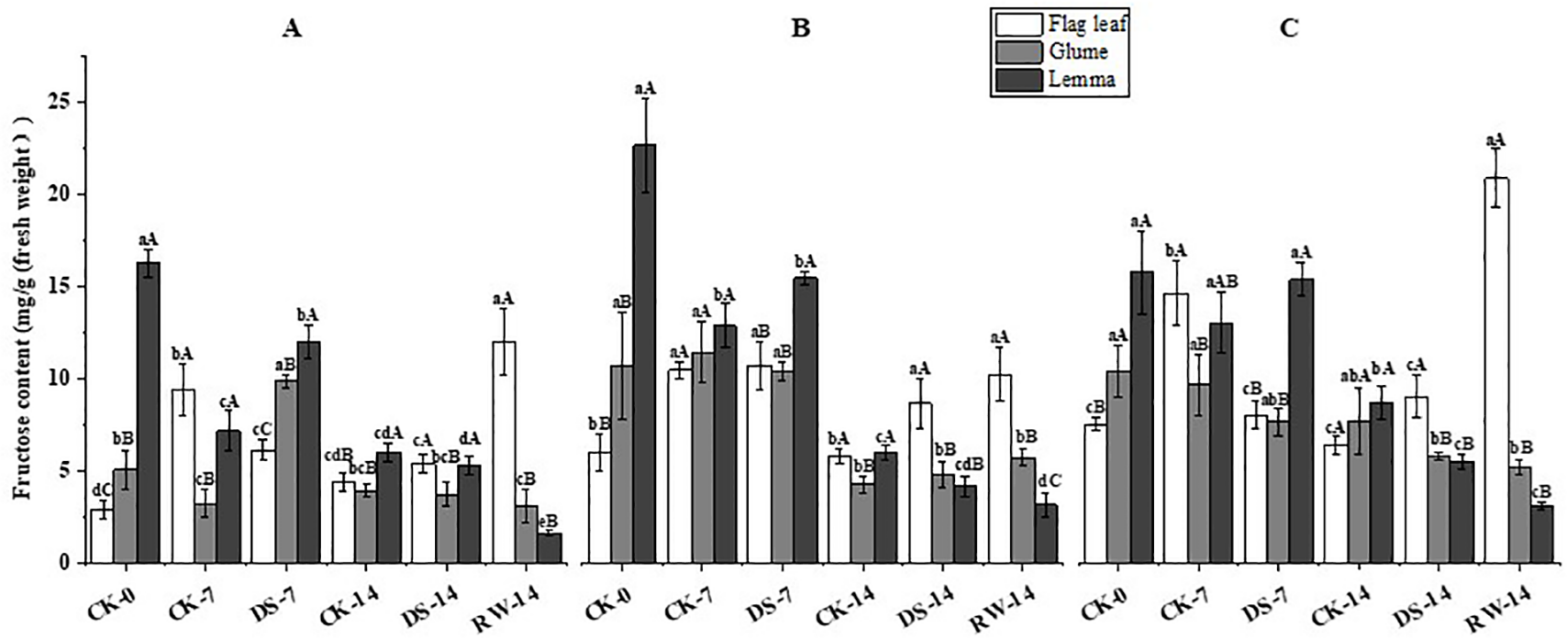
Fructose content in flag leaves, glumes, and lemmas under different watering treatments at different stages at three time periods: morning **(A)**, noon **(B)**, and evening **(C)**. Different lowercase letters within the same organ and different capital letters within the same treatment indicate a significant difference at the p < 0.05 probability level.

The fructose content of flag leaves was significantly (p < 0.05) lower under the DS-7 treatment than that under CK-7 in the morning and evening, while drought stress significantly (p < 0.05) increased the fructose content of glumes and lemmas in the morning (**Figure 6**). Compared to CK-14, the fructose content of flag leaves was significantly (p < 0.05) higher under DS-14 in the noon, but drought stress significantly (p < 0.05) decreased that of lemmas in the evening. Compared to DS-14, the fructose content of flag leaves was significantly (p < 0.05) higher under RW-14 in the morning and evening, while it was significantly lower in the lemmas in the morning. The lemmas had a significantly (p < 0.05) higher fructose content than the flag leaves under the CK-0 and DS-7 treatment in all three sampling times. Flag leaves had a significantly (p < 0.05) higher fructose content than both glumes and lemmas under CK-7 in the morning, under DS-14 in the noon and evening, and under RW-14 at all three sampling times.

**Figure 7:**
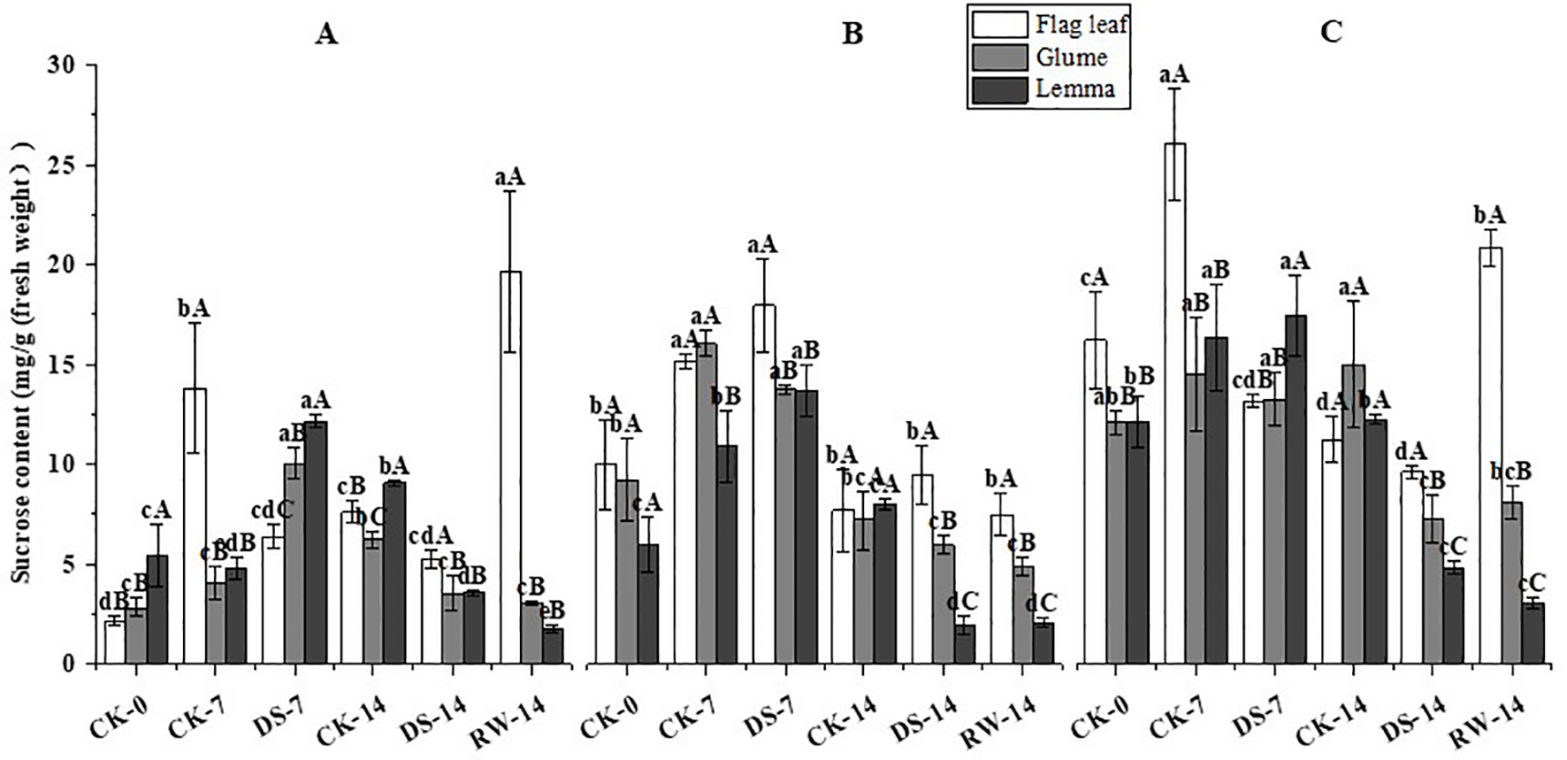
Sucrose content in flag leaves, glumes, and lemmas under different watering treatments at different stages at three time periods: morning **(A)**, noon **(B)**, and evening **(C)**. Different lowercase letters within the same organ and different capital letters within the same treatment indicate a significant difference at the p < 0.05 probability level.

The sucrose content of flag leaves was significantly (p < 0.05) lower under DS-7 than that under CK-7 in the morning and evening, while the sucrose content was significantly higher than that of lemmas and glumes in the morning and that of lemmas in the noon (**Figure 7**). The sucrose content of lemmas and glumes was significantly (p < 0.05) lower under DS-14 in the morning and evening compared to CK-14. The sucrose content of flag leaves was significantly (p < 0.05) higher under RW-14 than under DS-14 in the morning and evening, and the sucrose content was significantly (p < 0.05) lower than that of lemmas in the morning. Flag leaves had a significantly (p < 0.05) higher sucrose content than glumes and lemmas under CK-0 in the evening, under DS-7 in the noon, and under RW-14 at three sampling times. Lemmas had a significantly (p < 0.05) higher sucrose content than flag leaves and glumes under CK-0 in the morning, under DS-7 in the morning and evening, and under CK-14 in the morning.

**Figure 8:**
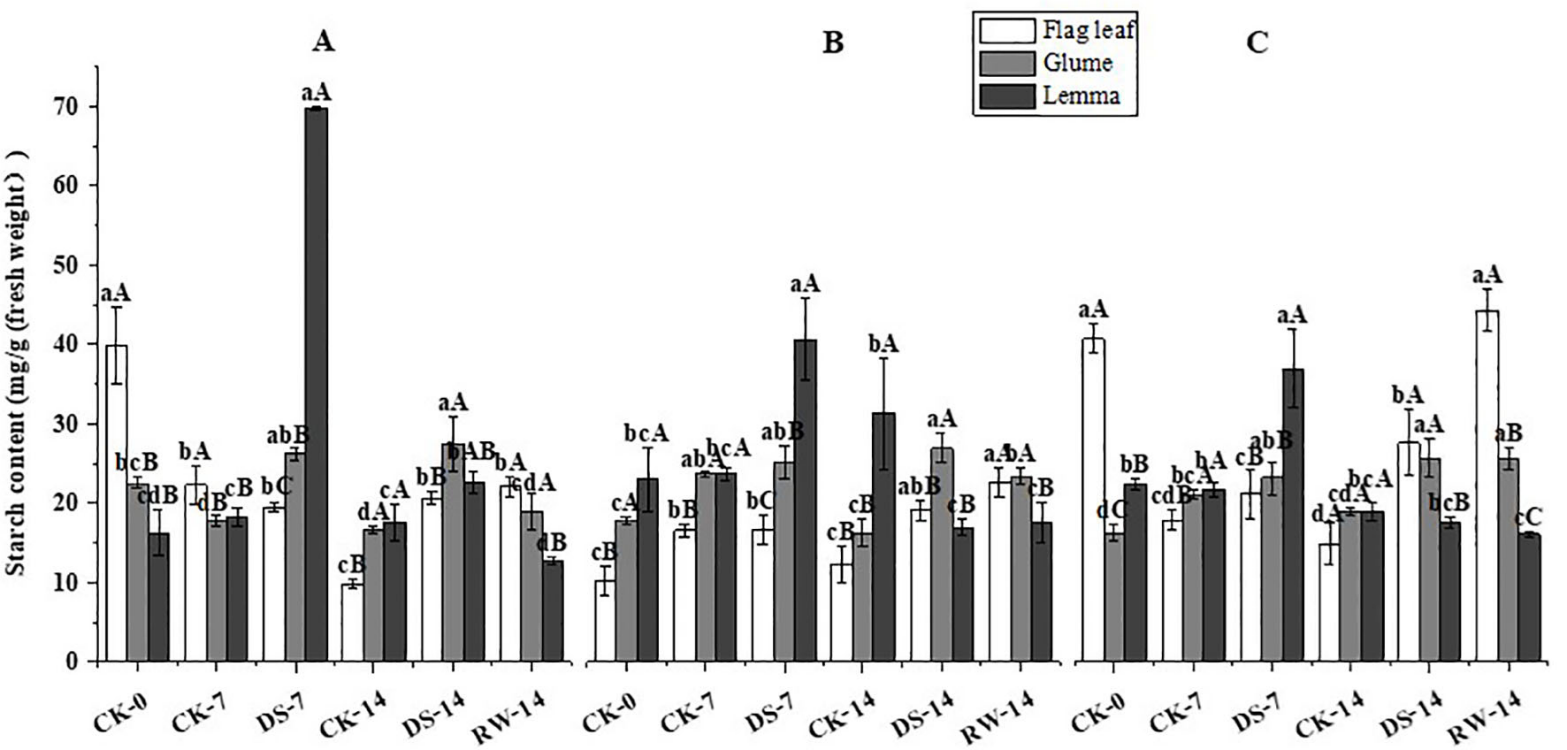
Starch content of flag leaves, glumes, and lemmas under different watering treatments at different stages at three time periods: morning **(A)**, noon **(B)**, and evening **(C)**. Different lowercase letters within the same organ and different capital letters within the same treatment indicate a significant difference at the p < 0.05 probability level.

Compared to CK-7, the starch content of glumes and lemmas was significantly (p < 0.05) higher under DS-7 in the morning, and that of lemmas was significantly (p < 0.05) higher under DS-7 in the noon and evening (**Figure 8**). Compared with CK-14, the starch content of flag leaves and glumes was significantly (p < 0.05) higher under DS-14 at all three sampling times. Additionally, lemma starch content was significantly (p < 0.05) higher under DS-14 in the morning and decreased significantly (p < 0.05) in the noon, compared to that under CK-14. Compared to DS-14, flag leaf starch content increased significantly (p < 0.05) under RW-14 in the evening, while the glume starch content decreased significantly (p < 0.05) under RW-14 in the morning and evening. At all three sampling times, the flag leaves starch content was significantly (p < 0.05) higher than that of glumes and lemmas under CK-0 in the morning and evening, CK-7 in the morning, and RW-14 in the evening. Lemmas had significantly (p < 0.05) higher starch content than flag leaves and glumes under DS-7 at three sampling times, and under CK-14 at noon. Under DS-14, glume starch content was significantly (p < 0.05) higher than that of the flag leaves and lemmas in the morning.

**Figure 9:**
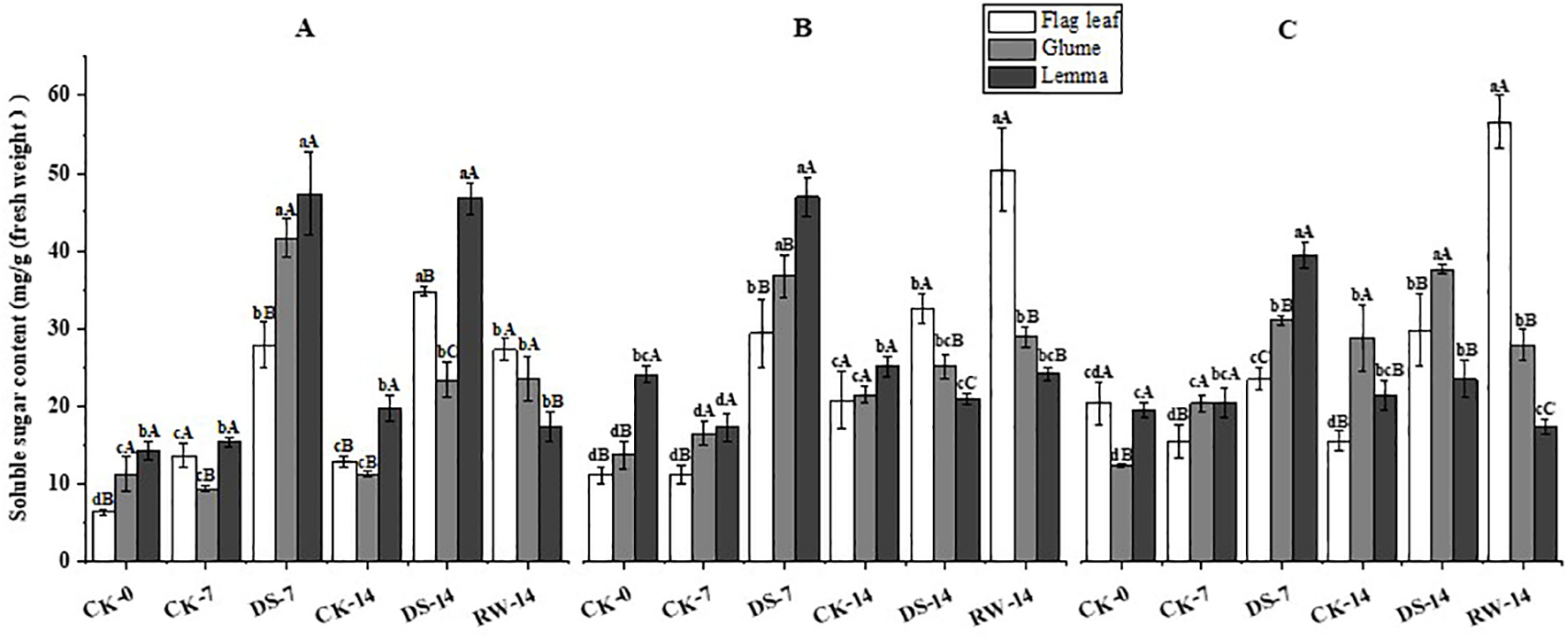
Soluble sugar content in flag leaves, glumes, and lemmas under different watering treatments at different stages at three time periods: morning **(A)**, noon **(B)**, and evening **(C)**. Different lowercase letters within the same organ and different capital letters within the same treatment indicate a significant difference at the p < 0.05 probability level.

Compared to CK-7, the soluble sugar content of flag leaves, glumes, and lemmas was significantly (p < 0.05) higher under DS-7 at the three sampling times (**Figure 9**). Compared to CK-14, the soluble sugar content of flag leaves and glumes in the morning and evening, as well as that of lemmas in the morning, was significantly (p < 0.05) higher under DS-14. The soluble sugar content of lemmas was significantly (p < 0.05) higher under DS-14 than under CK-14 in the morning, while it was significantly (p < 0.05) lower at noon. Compared to DS-14, the soluble sugar content of flag leaves was significantly (p < 0.05) lower in the morning and evening, and significantly higher in the noon in RW-14. The soluble sugar content of lemmas was significantly (p < 0.05) lower under RW-14 in the morning and evening, compared to that under DS-14. Compared to that under DS-14, the glume soluble sugar content was significantly (p < 0.05) lower under RW-14 in the evening. At all three sampling times, the soluble sugar content of flag leaves was significantly (p < 0.05) lower than that of the glumes and lemmas under CK-0 in the morning, under CK-7 in the noon and evening, and under DS-7 at all three sampling times, while it was significantly (p < 0.05) higher than that of glumes and lemmas under RW-14 in the noon and evening. Lemmas had a significantly (p < 0.05) higher soluble sugar content than flag leaves and glumes under DS-7 in the evening, and under CK-14 and DS-14 in the morning.

### Total C and N contents

**Figure 10:**
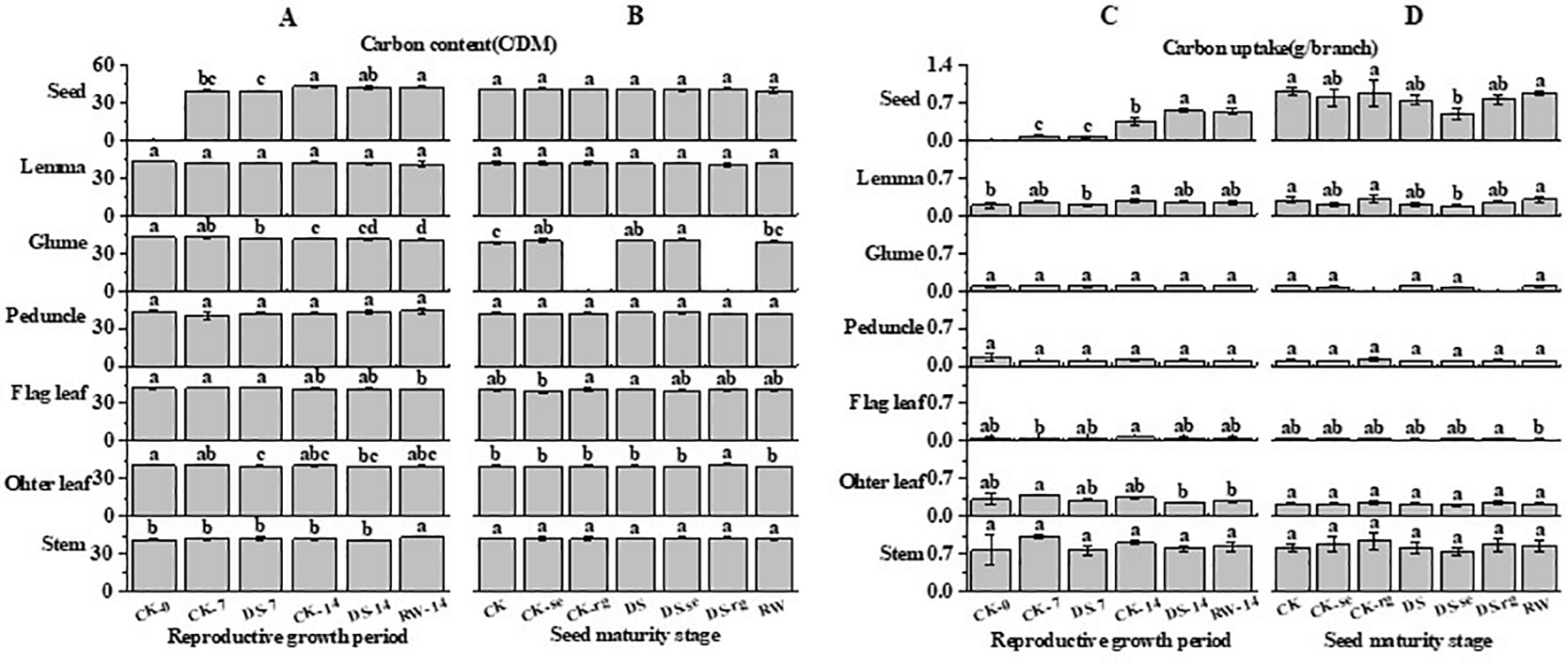
The C content **(A, B)** and C uptake **(C, D)** in stems, other leaves, flag leaves, peduncles, glumes, lemmas, and seeds during the reproductive growth period **(A, C)** and the seed maturity stage **(B, D)** under different watering treatments. Different lowercase letters within the same organ at the same stage indicate a significant difference at the p < 0.05 probability level.

As growth continued, the C content gradually decreased in the flag leaves, other leaves, and glumes, while the C content of the seeds gradually increased (**Figure 10**). Compared to that under CK-7, the C content of other leaves was significantly (p < 0.05) lower under the DS-7 treatment. During the reproductive growth period, the C content of the peduncles and lemmas was stable. At the seed maturity stage, the C content of the glumes was significantly higher under DS than that under CK.

As growth continued, the C uptake gradually decreased in other leaves, while it gradually increased in seeds (**Figure 10**). Compared to that under CK-14, the C uptake in seeds was significantly higher under DS-14.

**Figure 11:**
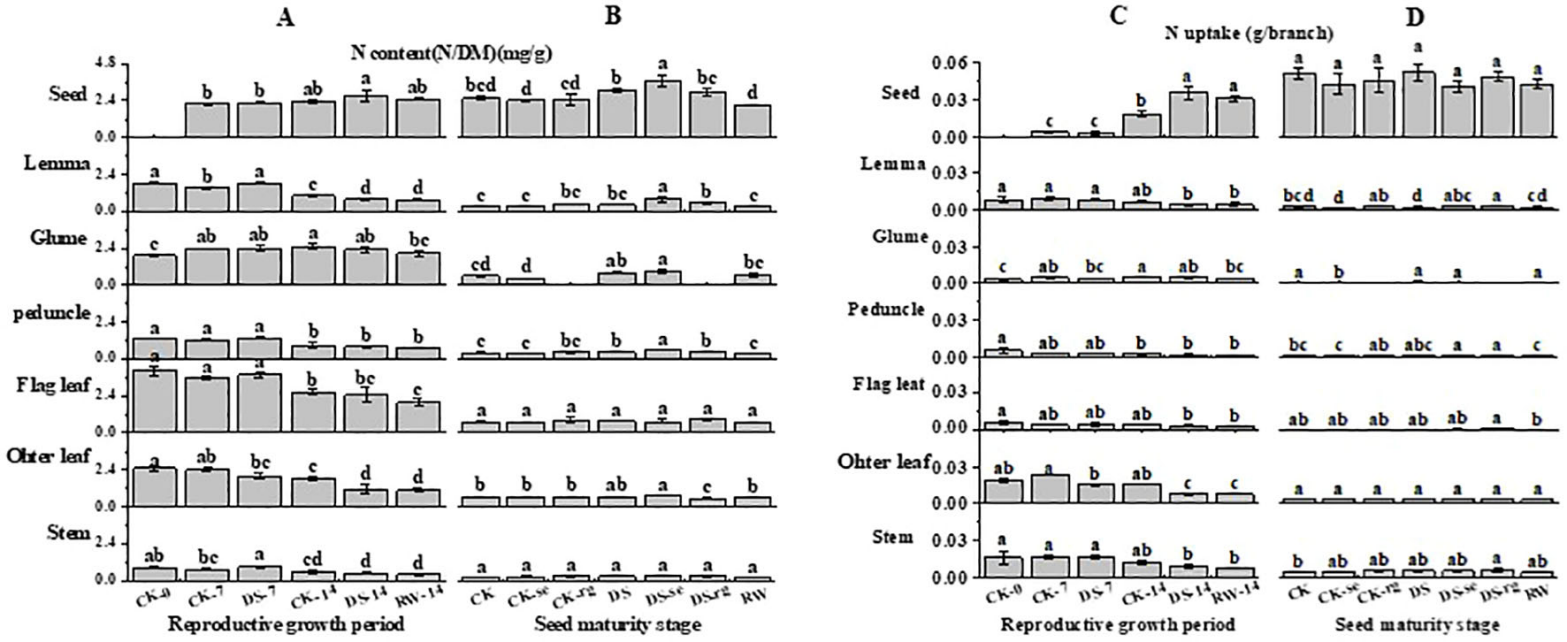
The N content **(A, B)** and N uptake **(C, D)** in stems, other leaves, flag leaves, peduncles, glumes, lemmas, and seeds during the reproductive growth period **(A, C)** and the seed maturity stage **(B, D)** under different watering treatments. Different lowercase letters within the same organ at the same stage indicate a significant difference at the p < 0.05 probability level.

As growth continued, the N content gradually decreased in the flag leaves, other leaves, stems, peduncles, and lemmas, while it first increased and then decreased in the glumes (**Figure 11**). Compared to CK-7, the N content of the lemmas and peduncles was significantly (p < 0.05) higher than that under DS-7. The N content of the lemmas and other leaves was significantly (p < 0.05) lower under DS-14, compared to that under CK-14. At the seed maturity stage, the DS leaves had a significantly (p < 0.05) higher N content in the glumes and peduncles than that in the CK leaves. Compared to DS, DS-se significantly (p < 0.05) increased the N content in the lemmas, peduncles, and seeds. The N content in the peduncles and seeds was significantly (p < 0.05) lower under RW than that under DS.

As growth continued, the N uptake in the lemmas, peduncles, flag leaves, other leaves, and stems gradually decreased, while it gradually increased in seeds (**Figure 11**). The N uptake in the glumes first gradually increased and then decreased with further growth. Compared to CK-7, other leaves had significantly (p < 0.05) lower N uptake under DS-7. Compared to CK-14, the N uptake in other leaves was significantly (p < 0.05) lower than that under DS-14, while drought stress significantly (p < 0.05) increased the N uptake in the seeds. At the seed maturity stage, CK-se significantly (p < 0.05) decreased the N uptake in the glumes compared to CK. DS-rg significantly increased the N uptake in lemmas compared to DS.

The C/N values gradually increased in stems, other leaves, flag leaves, peduncles, and lemmas with the furtherance of growth, while gradually decreasing in glumes (**Table 1**). Seeds had a stable C/N status. Compared to CK-14, the C/N values in other leaves was significantly (p < 0.05) higher under the DS-14 treatment. The C/N values in the stems, peduncles, and lemmas were significantly (p < 0.05) higher under RW-14 compared to those under DS-14.

**Table 1:** The C/N values in the stems, other leaves, flag leaves, peduncles, glumes, lemmas, and seeds during the reproductive growth period and the seed maturity stage under different watering treatments.

Stage	Treatment	Stem	Other leaf	Flag leaf	Peduncle	Glume	Lemma	Seed
Reproductive growth period	CK-0	50.0 ± 3.1d	16.5 ± 1.1b	10.5 ± 0.6d	33.3 ± 1.0a	22.0 ± 0.6a	23.3 ± 0.8d	——
	CK-7	62.0 ± 5.9cd	17.1 ± 0.6b	11.8 ± 0.5cd	32.2 ± 0.8a	18.1 ± 0.3bc	27.8 ± 1.3cd	18.2 ± 0.2a
	DS-7	48.6 ± 8.0d	20.0 ± 1.6b	11.2 ± 0.6cd	30.6 ± 2.5a	17.7 ± 1.2bc	22.9 ± 0.8d	18.2 ± 0.2a
	CK-14	79.2 ± 13.1bc	22.0 ± 1.3b	15.6 ± 0.9bc	48.1 ± 4.7b	16.5 ± 1.0c	42.4 ± 2.3bc	18.3 ± 1.0a
	DS-14	90.5 ± 10.4b	37.8 ± 9.2a	17.7 ± 3.6ab	52.5 ± 3.4b	17.9 ± 1.3bc	54.0 ± 4.2b	15.6 ± 2.5a
	RW-14	113.6 ± 13.6a	38.8 ± 4.5a	21.0 ± 2.7a	60.6 ± 2.6a	19.7 ± 1.8ab	75.8 ± 19.6a	16.9 ± 0.4a
Seed maturity stage	CK	219.4 ± 24.3a	66.5 ± 4.5b	59.2 ± 7.8a	122.6 ± 15.4a	64.6 ± 8.0b	123.4 ± 18.5a	16.1 ± 1.0abc
	CK-se	202.0 ± 12.7ab	62.9 ± 3.6b	55.3 ± 2.0a	118.9 ± 6.8a	87.2 ± 5.7a	114.5 ± 6.8ab	17.0 ± 0.4ab
	CK-rg	182.9 ± 41.5ab	66.5 ± 6.0b	53.0 ± 11.1a	105.4 ± 10.4ab	——	91.5 ± 9.7bc	17.1 ± 2.0ab
	DS	167.9 ± 10.7ab	61.5 ± 3.0b	52.8 ± 2.0a	95.5 ± 0.9b	51.8 ± 5.1bc	99.2 ± 3.6b	13.3 ± 0.7cd
	DS-se	145.8 ± 22.3b	55.3 ± 2.3b	56.2 ± 10.4a	74.6 ± 2.0c	47.5 ± 9.2c	59.0 ± 12.7d	11.1 ± 1.3d
	DS-rg	163.7 ± 44.5ab	80.2 ± 9.0a	47.5 ± 2.1a	92.8 ± 10.5bc	——	74.0 ± 8.2cd	14.1 ± 1.4bc
	RW	209.3 ± 11.7ab	65.8 ± 0.5b	58.8 ± 1.1a	119.4 ± 5.8a	61.2 ± 6.1bc	132.8 ± 4.6a	19.2 ± 1.8a

Different lowercase letters within the same organ during the reproductive growth period or the seed maturity stage in the same column indicate a significant difference at the p < 0.05 probability level.

At the seed maturity stage, the C/N values in the peduncles and lemmas under DS were significantly (p < 0.05) lower than those under CK (**Table 1**). The C/N values in the peduncles, lemmas, and seeds under RW were significantly (p < 0.05) higher than those under DS. The C/N values in the glumes under CK were significantly (p < 0.05) higher compared to those under CK-se. Compared to those under DS, the C/N in other leaves were significantly (p < 0.05) higher under DS-rg, while that in lemmas significantly (p < 0.05) decreased.

### Dry matter mass

**Figure 12:**
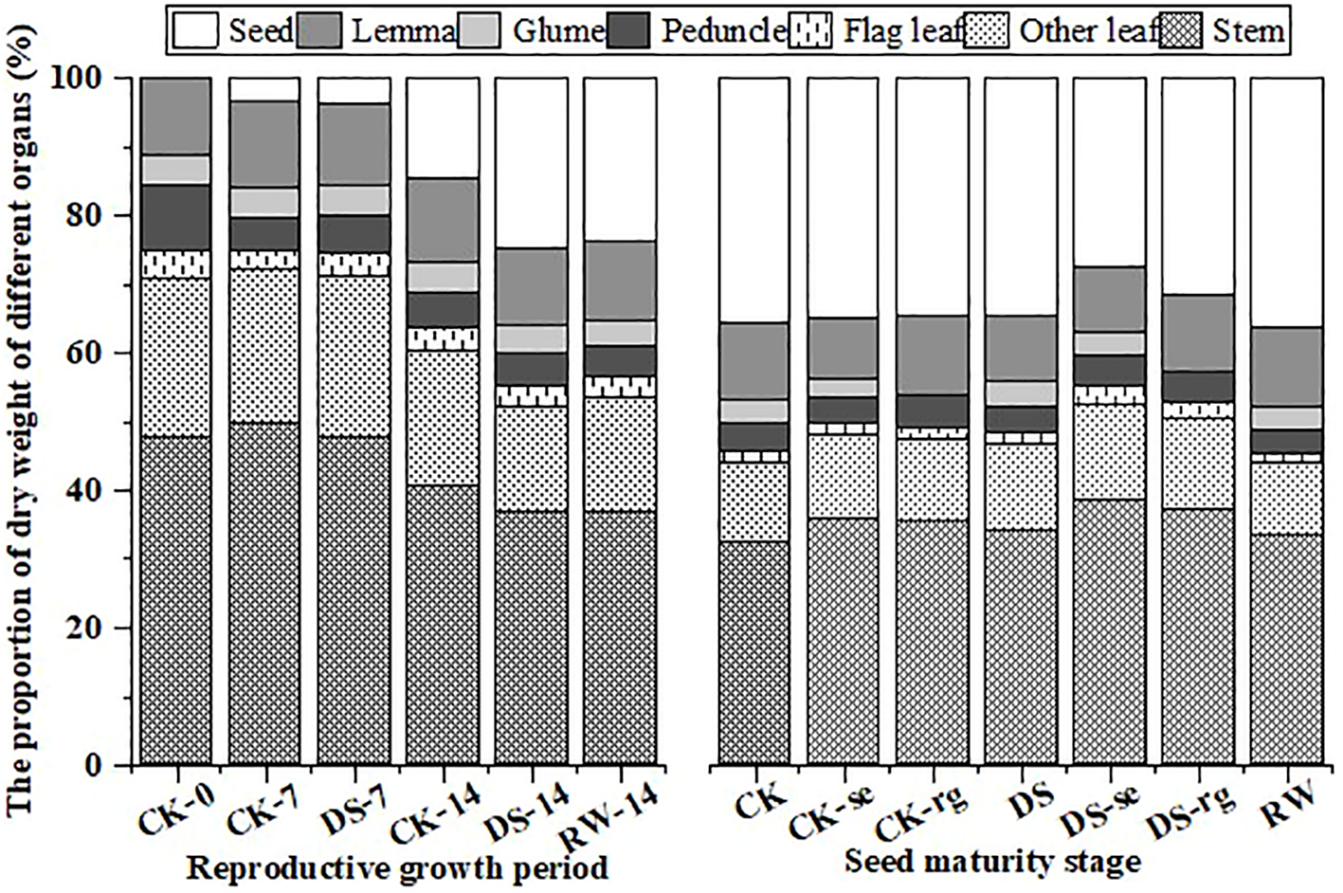
The proportion of dry matter mass in the stems, other leaves, flag leaves, peduncles, glumes, lemmas, and seeds during the reproductive growth period and the seed maturity stage under different watering treatments.

As growth continued, the mass proportion of the stems, other leaves, flag leaves, and peduncles gradually decreased, while the mass proportion of the glumes and lemmas remained stable (**Figure 12**). The weight proportion of seeds gradually increased with the continuation of growth, and drought stress promoted dry matter accumulation in the seeds. Under drought stress, DS-se and DS-rg decreased a greater proportion of seed weight than CK-se and CK-rg.

**Figure 13:**
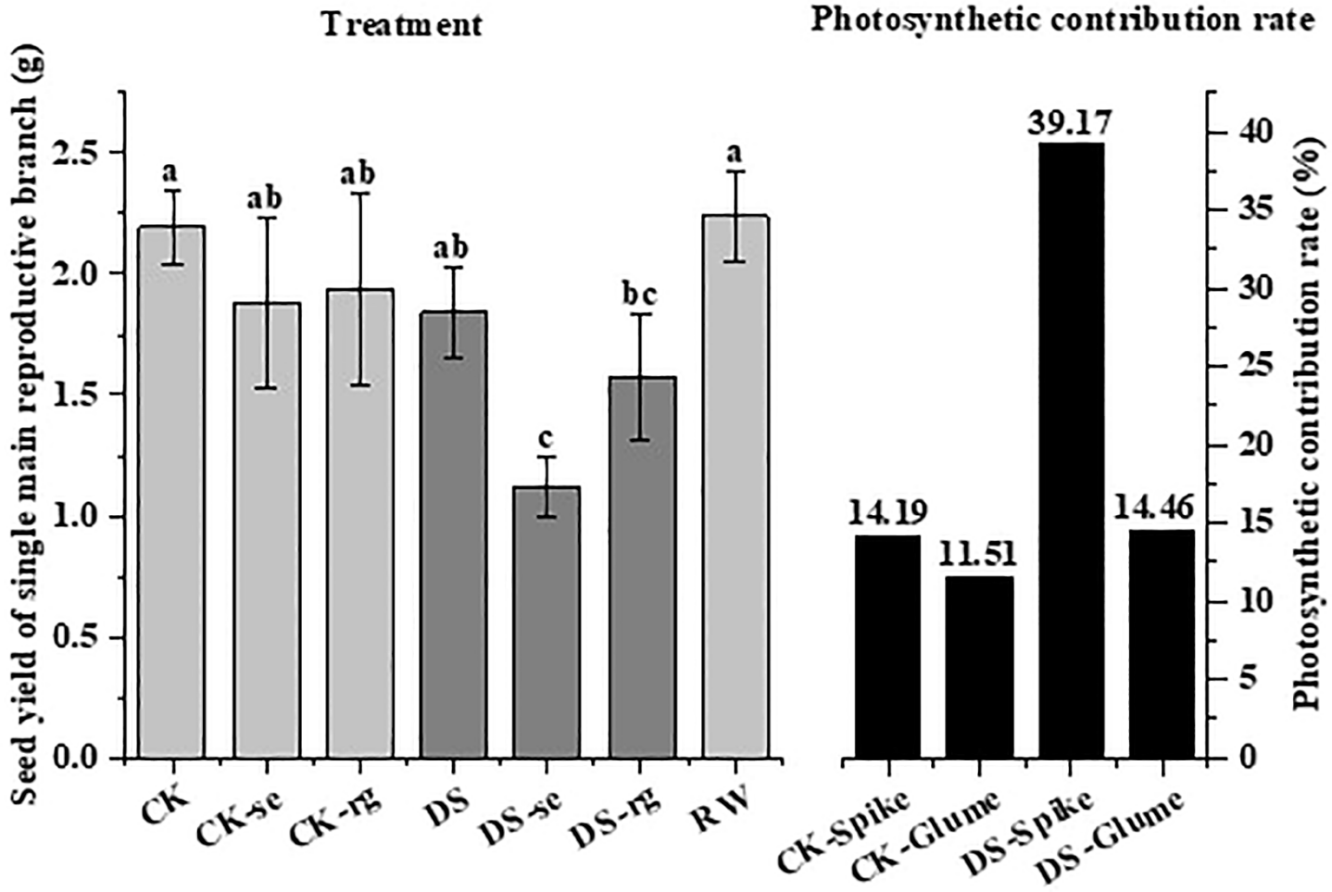
The seed yield under different watering treatments and the photosynthetic contribution rate of the ears and glumes under different watering treatments. Different lowercase letters indicate a significant difference at the p < 0.05 probability level.

There was no significant difference in seed yield under CK, CK-se, or CK-rg, while the seed yield under DS-se was significantly (p < 0.05) lower than that under DS (**Figure 13**). Drought stress increased the ears’ photosynthetic contribution to seed yield.

## Discussion

### Water status response to drought stress

The water status of a plant is related to organ types, growth stages, and environmental conditions. Drought reduces plants’ water content and accelerates their senescence (Tan et al., 2023). In this study, drought stress significantly (p < 0.05) decreased the AWC in flag leaves, while having no significant effect on the AWC of glumes and lemmas (**Figure 2**). In terms of cell structure, the outer epidermis of the glumes and lemmas was lignified, and the stomata were degraded (**Figure 1**). The inner epidermal stomata had smaller stomatal chambers. They are less affected by adverse environmental conditions in terms of stomatal conductance (Tambussi et al., 2005) and therefore have lower transpiration rates than the flag leaves. Glumes and lemmas have a thick epidermis and cuticle on their outer sides helping maintain expansion pressure and facilitate gas exchange under drought stress (Tambussi et al., 2005). These structural characteristics also provide the ears with a higher relative water content and greater osmotic adjustment (Rao and Chaitanya, 2016). Moreover, in this study, compared to DS-14, the AWC of flag leaves under the RW-14 treatment was significantly (p < 0.05) higher, and this result may be related to the compensatory effect of drought rehydration in plants (Li et al., 2024). Plants exhibit a high osmotic adjustment capacity for an extended period after rehydration facilitating rapid leaf growth and compensating for the losses caused by drought (Su and Shan, 1995). In addition, the ear organs might possess higher osmotic adjustment ability under drought conditions (Tambussi et al., 2005), since drought stress induces higher grain filling speed and vast soluble nutrient transfer to grains through other ear organs contributing to maintaining water status (Rao and Chaitanya, 2016). Thus, stable water status in the ear organs is a critical part of maintaining physiological performance when drought occurs.

### Photosynthetic performance under drought stress

Rubisco is the key enzyme responsible for fixing CO_2_, participating in the Calvin cycle. It also catalyzes the oxidation of photorespiratory carbon and serves as the primary receptor of CO_2_. Drought stress decreases Rubisco activity, which affects plant photosynthetic ability by limiting the Calvin cycle (Jia et al., 2015; Wang et al., 2020). The present study also reached a similar conclusion: the Rubisco activities in flag leaves, glumes, and lemmas showed varying degrees of decline under DS-7 compared to those under CK-7 (**Figure 3**). Rubisco activity in ear organs is less affected by drought stress (Simova-Stoilova et al., 2020; Zhang et al., 2019; Ding et al., 2018). Rubisco might not only act in C fixation but also play an important role in N metabolism in ear organs (Lopes et al., 2006). The proportion of Rubisco in soluble protein is 30%–60%, which can be mobilized and plays important roles under adverse situations (Feller et al., 2008). Lopes et al. reported that Rubisco levels were much greater in the glumes of wheat ears than in flag leaves during the milk to early dough stages (Lopes et al., 2006). Rubisco acts as the main storage protein in ear organs and maybe even more important than flag leaves in grain filling during the final development stages, especially when facing stress. Glumes can act as a temporal sink for N in the absence of an external fertilizer. During grain filling, glumes are converted into an N source presumably remobilizing their accumulated nitrogen to the grains (Lopes et al., 2006; Waters et al., 1980). Approximately 23% of the mature seed grain’s contribution comes from the glumes (Simpson et al., 1983).

It has been found that C4 photosynthetic enzyme activities are elevated in ear organs under drought stress (Jia et al., 2015). Gene expression of PEPC is repressed in flag leaves but is induced such that it is highly expressed in ear organs (Zhang et al., 2019; Vicente et al., 2018). In this study, although drought stress significantly decreased PEPC activity in the flag leaves, glumes, and lemmas, the glumes were able to maintain higher PEPC activity under drought stress in the later growth stage compared to the flag leaves and lemmas (**Figure 4**). PEPC provides carbon skeletons for biosynthesis by fixing internally released CO_2_ and assimilating N helping plants withstand drought stress. After rewatering, PEPC activity in both the glumes and lemmas significantly rebounded, but this was not observed in the flag leaves. This result reflects the fact that ear organs play an important role in photosynthesis during the late growth period by compensating for the reduction in the photosynthetic ability of flag leaves (Tian et al., 2022).

The process of photosynthesis in plants is influenced not only by the external environment but also by a plant’s growth and development, metabolic activity, and other factors (Sukhov, 2016). By plotting a light response curve, physiological parameters, such as the maximum photosynthetic rate, dark respiration rate, light saturation point, and light compensation point, of plants can be obtained. In this study, as light intensity increased, the photosynthetic rate of all organs increased and that of the flag leaf was significantly higher than that of various ear organs (**Figure 5**). The flag leaves serve as the primary photosynthetic organ of the plant (Brazel and Ó Maoiléidigh, 2019) featuring highly optimized physiological structures and functions to maximize light capture and photosynthetic efficiency. The flag leaf typically exhibits high sensitivity to changes in light intensity allowing it to adjust its photosynthetic rate in response to increasing or decreasing light intensity to adapt to different environmental conditions. Conversely, ear organs are less sensitive to changes in light intensity compared to the flag leaves, and their photosynthetic rate exhibits a relatively smaller range of variation (**Figure 5**). At lower light intensities, the photosynthetic rate of ear organs is more severely limited, constrained by their physiological structures and functions, including factors such as a lower number of stomata (**Figure 1**) and reduced chlorophyll levels (Tian et al., 2022). When reaching the maximum light intensity, the flag leaves, glumes, and peduncles did not exhibit photoinhibition, while the lemmas showed photoinhibition (**Figure 5**). The photoinhibition of lemmas may be attributed to the fact that glumes cover lemmas, which always receive a low level of light. In addition, lemmas lacks adequate photoprotective mechanisms to cope with high light intensities, such as weaker regulatory abilities via photorespiration and other pathways (Zeng et al., 2024). The photosynthetic characteristics of lemmas are similar to those of shade plants, including stomatal closure and photoinhibition under intense light (Martinez et al., 2003). Under strong light or sufficient CO_2_ supplementation, the photosynthetic rate per unit mass of flag leaves was higher than that of ear organs, indicating that leaves are the main photosynthetic organs (Brazel and Ó Maoiléidigh, 2019). The photosynthetic rates per unit mass of flag leaves and glumes decreased with reduced light intensity at a similar rate (**Figure 5**). This suggests that the photosynthesis-limiting factors of flag leaves and glumes were essentially the same under low light conditions at 300 µmol m^−2^ s^−1^. The plant CO_2_ response curve provides deeper insights into the photosynthetic mechanism of plants and is an important indicator for assessing their photosynthetic capacity (Li et al., 2020). In this study, the lemmas reached the CO_2_ compensation point first, and its photosynthetic rate was significantly lower than that of the flag leaves and glumes. At CK-0, the photosynthetic rate of the lemmas was limited. With the enhancement of metabolic activities within the plant, the respiratory rate increased relatively. In addition, shading by the glumes and limitations in gas exchange further affected the lemmas making it more prone to reaching the CO_2_ compensation point. When the CO_2_ concentration reached from 600 µmol m−¹ to the CO_2_ saturation point, the photosynthetic rate of the flag leaves was significantly higher than that of the ear organs, since the flag leaves had a higher stomatal density (Tian et al., 2022), which facilitates efficient gas exchange, including the absorption of CO_2_ and the release of O_2_.

### Carbon metabolism and allocation under drought stress

Non-structural carbohydrates, representing the outcome of photosynthesis, serve as both nutrients and regulators of plant growth and development. They are crucial for osmotic adjustment (Ruan, 2014), the induction of resistance gene expression (Jia et al., 2013), antioxidant regulation (Tauzin and Giardina, 2014), and tissue differentiation and development (Ohto et al., 2001; Iraqi and Tremblay, 2001) under abiotic stresses. One of these carbohydrates, fructose, is an intermediate product of photosynthetic C metabolism, while sugar metabolism and sucrose serve as the primary form for exporting and translocating photosynthetic products (Ruan, 2012). In this study, DS-7 significantly decreased the fructose and sucrose content in flag leaves in the morning and evening, while significantly increasing the carbohydrate content in both glumes and lemmas, compared to CK-7 (**Figures 6**, **7**). When exposed to drought conditions, an imbalance will be generated between carbohydrate production and utilization in the flag leaves. Previous studies reported that sucrose synthase and sucrose phosphate synthase activities were significantly greater in the seedlings of a tolerant wheat cultivar than in sensitive ones under drought stress conditions (Nemati et al., 2018). Drought-tolerant wheat genotypes have more effective strategies for fructan remobilization and sucrose synthesis and transport than drought-sensitive genotypes because they can maintain higher levels of photosynthesis (Bagherikia et al., 2019). In addition, RW-14 could significantly increase flag leaves’ fructose and sucrose content, while the variation in glumes and lemmas was lower than that in flag leaves (**Figures 6**, **7**). Ear organs showed higher photosynthetic stress resistance and were able to maintain a more stable C metabolism than leaves contributing to grain filling (Tian et al., 2022; Jia et al., 2015). Furthermore, the accumulation of soluble sugars, including fructose and sucrose, promotes osmotic adjustment in ear organs when drought occurs (Xiao et al., 2024). In this study, at all three sampling times, drought stress significantly increased the soluble sugar content in glumes, lemmas, and flag leaves, and that in ear organs was significantly higher (**Figure 9**). Leaves experienced a more moderate increase in soluble sugar content under drought conditions (Zhao et al., 2024). This suggests that ear organs exhibited better tolerance to drought stress adapting to this water-deficient environment by producing more soluble materials. Starch is one of the primary forms of energy storage in plant organs. A drought might accelerate the rate of transfer of photosynthetic assimilates to the pool organs. Once transferred, these assimilates undergo a series of catabolic–synthetic reactions and are ultimately stored in the pool organs as starch (Abdelgawad et al., 2020). In this study, DS-7 significantly increased the starch content in glumes and lemmas in the morning and evening, and glumes and lemmas had a higher starch content than flag leaves (**Figure 8**). On the one hand, the large accumulation of fructose and sucrose contributed to the synthesis of starch. On the other hand, besides acting as the source organs, ear organs also temporarily store the photosynthetic assimilates adjacent to the seeds. The transformation of starch to soluble sugars in ear organs could enhance the osmoregulatory capacity of the ears and promote the transfer of nutrients to seeds ensuring the normal growth and development of a plant while enabling it to resist adverse stresses.

Drought reduces the accumulation of photosynthetically active substances in plants and increases respiratory consumption, thereby reducing plant dry matter accumulation (Plaut et al., 2004). In this study, under drought stress, the dry matter of all organs gradually shifted to seeds as growth continued, and the percentages of stems and leaves decreased significantly (**Figure 12**) aligning with the findings of Abid et al. (2018). This result may reflect the transfer of non-structural carbohydrates to the developing seeds. In this study, oat ears were subjected to ear shading and glume removal at the initial flowering period. It was observed that both ear shading and glume removal decreased oat seed yield (**Figure 13**). The reduction in seed yield was larger under drought stress implying that drought stress increases the photosynthetic contribution of ear organs to grain filling aligning with the findings of Wang et al. (2016).

## Conclusion

In this study, we evaluated the changes in absolute water content, photosynthetic enzyme activities, non-structural carbohydrates, total carbon and nitrogen contents, and dry matter accumulation in the flag leaves, glumes, and lemmas of oat plants under drought stress. The results indicated that drought had no significant impact on the absolute water content and Rubisco enzyme activity in the glumes and lemmas. Furthermore, during the late grain-filling stage, the PEPC activity in the glumes remained at a relatively high level. Drought stress significantly increased the contents of fructose, sucrose, starch, and soluble sugars in the spike organs enabling them to exhibit higher photosynthetic performance and better osmotic adjustment capabilities. As the seeds developed and grew, carbon, nitrogen, and dry matter accumulated gradually, with the spike organs serving as storage organs for C and N metabolites. In conclusion, oats tolerates drought maintaining a high AWC without ear physiological functions and yield. During drought stress, oat ear organs contribute to maintain the seed yield by keeping water status and photosynthetic performance.

## Data Availability

The original contributions presented in the study are included in the article/supplementary material. Further inquiries can be directed to the corresponding author.
